# Toward Blood-Based Precision Medicine: Identifying Age-Sex-Specific Vascular Biomarker Quantities on Circulating Vascular Cells

**DOI:** 10.1007/s12195-023-00771-1

**Published:** 2023-07-06

**Authors:** Yingye Fang, Ling Chen, P. I. Imoukhuede

**Affiliations:** 1 Department of Bioengineering, University of Washington, Seattle, WA USA; 2 Division of Biostatistics, Washington University in St. Louis School of Medicine, St. Louis, MO USA

**Keywords:** VEGFR, Angiogenesis, Biomarker, Circulating endothelial cells, Circulating progenitor cells, Quantitative flow cytometry, Blood biopsy

## Abstract

**Introduction:**

Abnormal angiogenesis is central to vascular disease and cancer, and noninvasive biomarkers of vascular origin are needed to evaluate patients and therapies. Vascular endothelial growth factor receptors (VEGFRs) are often dysregulated in these diseases, making them promising biomarkers, but the need for an invasive biopsy has limited biomarker research on VEGFRs. Here, we pioneer a blood biopsy approach to quantify VEGFR plasma membrane localization on two circulating vascular proxies: circulating endothelial cells (cECs) and circulating progenitor cells (cPCs).

**Methods:**

Using quantitative flow cytometry, we examined VEGFR expression on cECs and cPCs in four age-sex groups: peri/premenopausal females (aged < 50 years), menopausal/postmenopausal females (≥ 50 years), and younger and older males with the same age cut-off (50 years).

**Results:**

cECs in peri/premenopausal females consisted of two VEGFR populations: VEGFR-low (~ 55% of population: population medians ~ 3000 VEGFR1 and 3000 VEGFR2/cell) and VEGFR-high (~ 45%: 138,000 VEGFR1 and 39,000–236,000 VEGFR2/cell), while the menopausal/postmenopausal group only possessed the VEGFR-low cEC population; and 27% of cECs in males exhibited high plasma membrane VEGFR expression (206,000 VEGFR1 and 155,000 VEGFR2/cell). The absence of VEGFR-high cEC subpopulations in menopausal/postmenopausal females suggests that their high-VEGFR cECs are associated with menstruation and could be noninvasive proxies for studying the intersection of age-sex in angiogenesis. VEGFR1 plasma membrane localization in cPCs was detected only in menopausal/postmenopausal females, suggesting a menopause-specific regenerative mechanism.

**Conclusions:**

Overall, our quantitative, noninvasive approach targeting cECs and cPCs has provided the first insights into how sex and age influence VEGFR plasma membrane localization in vascular cells.

**Supplementary Information:**

The online version contains supplementary material available at 10.1007/s12195-023-00771-1.

## Introduction

Dysregulated angiogenesis is a characteristic of many vascular diseases and cancers, and better biomarkers of abnormal vessels are needed to evaluate patients and therapies. Two vascular endothelial growth factor receptors, VEGFR1 and VEGFR2, are central regulators of angiogenesis and are biomarkers for many of these diseases. VEGFR3, on the other hand, is primarily expressed by lymphatic endothelial cells, regulates a different process called lymphangiogenesis, and is thus not investigated in this work. VEGFRs on the cell plasma membrane bind VEGF ligands and initiate downstream angiogenic signaling. The relative abundance of VEGFR1 and VEGFR2 on endothelial cell plasma membranes dictates vascular growth patterns: during sprouting angiogenesis, VEGFR1 is highly expressed by trailing endothelial cells (stalk cells), and VEGFR2 is highly expressed by leading endothelial cells (tip cells) [8, 10, 25] and is essential for driving angiogenic sprouting, which rarely occurs in healthy adult tissues. As such, VEGFRs are expressed at low levels in resting endothelial cells compared to angiogenesis-active conditions [47, 70].

The role of VEGFR1 is less understood compared to the well-established pro-angiogenic VEGFR2. VEGFR2 mediates pro-angiogenic hallmarks, including endothelial cell permeability, proliferation, migration, and survival in both physiological and pathological conditions [79]. VEGFR1 is conventionally described as a decoy receptor [69] because its affinity for VEGF is ten times higher than that of VEGFR2 [21, 53, 75], but the resulting angiogenic signaling does not initiate hallmark angiogenic activities, such as endothelial cell proliferation and migration in vitro [78]. Paradoxically, this decoy role of VEGFR1 does not appear to hold in many pathological conditions, since VEGFR1 overexpression has been correlated with active vascular growth in cancers, ischemic tissue, and obese adipose tissue expansion [13, 32, 43, 62]. Despite many efforts to translate VEGFR expression into a reliable indicator for vascular diseases, VEGFR biomarker studies are often qualitative or semiquantitative and thus, are difficult to compare when making VEGFR-driven predictions for personalized prognoses.

In pursuing quantitative vascular biomarkers, we first need to find ways to reliably detect angiogenic biomarkers and ultimately quantify them. We have optimized quantitative flow cytometry as a tool for plasma membrane receptor characterization [23] and applied it to several tyrosine kinase receptors (RTKs), including VEGFRs [34, 35], Platelet-Derived Growth Factor Receptors [15], Epidermal Growth Factor Receptor [17], Oxytocin Receptor [15, 51, 52], Axl [22], and others [15, 17, 23]. We also have quantified RTKs on several cell types, including healthy [35] and ischemic [33] endothelial cells from mouse skeletal muscle, ovarian tumor cell lines [22], endothelial cells isolated from breast tumor xenograft [36], and patient-derived glioblastoma xenografts [17]. Collectively, these quantitative studies showed that vascular pathologies, such as ischemic disease and cancers, are associated with dysregulated RTK concentrations, particularly the dysregulation of membranous VEGFRs. However, clinical data acquisition has been impeded by the limited access to human vessel biopsies, which require invasive procedures or expensive imaging modalities (e.g., PET and functional MRI) [30]. It is imperative to identify more accessible angiogenic cells to study vascular biomarkers in human patients.

Two alternative candidate noninvasive vascular targets, circulating progenitor cells (cPCs) and circulating endothelial cells (cECs), have expanded our knowledge of vascular pathology [56, 66] and have predictive and therapeutic values in various vascular diseases and cancers [5, 24, 26]. Both cECs and cPCs closely interact with vascular endothelium during blood vessel formation and remodeling: cECs are shed from vascular endothelium during the expansion or damage of blood vessels, [82] cPCs promote vessel growth by either merging with or interacting with vascular endothelium [3, 46, 60], and large quantities of VEGFR1^+^ or VEGFR2^+^ cPCs are found in tumor metastases [38, 74], suggesting that VEGFRs in cPCs are potentially useful as therapeutic biomarkers or therapeutic targets in metastatic cancers. Yet, most cEC and cPC studies have focused on cell enumeration and suggested that increased numbers of cECs [6, 19] or cPCs [2–4, 20, 38, 45, 67, 73, 74, 84, 85] are correlated with tumor angiogenesis or vascular diseases [7, 86]. However, cPCs and cECs have been overlooked as accessible indicators of VEGFR expression in the tissue endothelium. Here, we consider the use of both cPCs and cECs as reporters of the vascular expression of VEGFRs.

Developing VEGFR biomarkers starts with establishing baseline levels of plasma membrane VEGFRs in healthy subjects, taking into account natural variations due to physiological angiogenic processes, such as aging and menstruation. Here, we present the design of a standardized workflow for isolating cECs and cPCs from blood and characterizing VEGFR1 and VEGFR2 localization on plasma membranes. Using this workflow, we established healthy baselines for VEGFR concentrations in cPCs and cECs. Further, because angiogenic capacities are known to vary by age [44], sex [64], race [55], and menopausal status [49], we examined whether these characteristics were correlated with VEGFR concentrations on cPCs and cECs. Lastly, we were able to differentiate and characterize heterogeneous subpopulations of cECs according to their VEGFR expression levels, specifically VEGFR-high and VEGFR-low. Our approach and measurements are foundational for advancing the VEGFR-driven prognosis of vascular diseases.

## Results

### Blood-Based Proteomic Analysis Workflow Establishes VEGFR Quantities on Circulating Vascular Cells

We designed a four-step workflow: (1) cPC/cEC enrichment and stratification, (2) plasma membrane VEGFR quantification, (3) statistical analysis of the correlation between subject characteristics (such as age, sex, race, and menopausal status) and inter-sample variations, and (4) mixture modeling analysis for dissecting intra-sample heterogeneity.

**Fig. 1:**
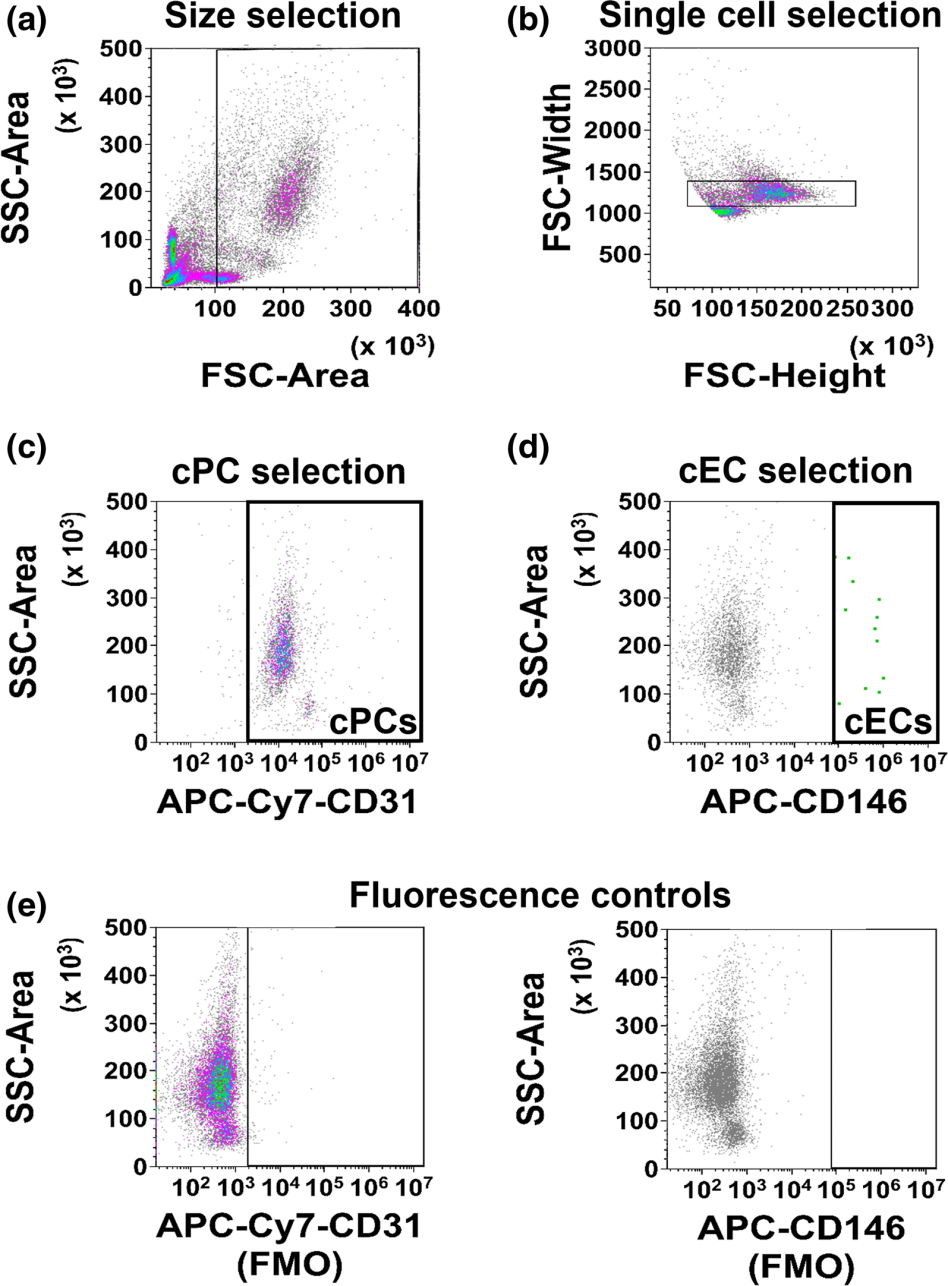
Circulating progenitor cells (cPCs) and circulating endothelial cells (cECs) were identified in the pre-enriched CD34 + cell population. **A** Debris and small platelets (≤ 6 µm) were excluded from the pre-enriched CD34 + cells. **B** Cell singlets were selected based on cell width (FSC-Width). **C** cPCs were identified as CD34 + CD31 + cells. **D** cECs were identified as CD34 + CD31 + CD146 + and were rare in the healthy blood samples, comprising 0.98 ± 0.29% of the cPC population (n = 23, mean ± SEM). **E** Fluorescence controls (i.e., FMO) of APC-Cy7-CD31 and APC-CD146 were used to set the respective positive gates

We obtained blood samples from 23 healthy subjects (6 menopausal/postmenopausal and 5 pre/peri-menopausal females and 12 age-matched males). Subject characteristics such as age, sex, and race are listed in Supp. Table 1. Subjects’ ages ranged from 25 to 60 years old, with a median of 51 years old and an interquartile range (IQR) of 18 years old (37–55 years old). The median and IQR are reported here due to the nonnormal age distribution of the samples (Shapiro–Wilk test p = 0.0156). The subjects were Black (non-Hispanic) (52.2%, n = 12), white (non-Hispanic) (26.1%, n = 6), and Hispanic (21.7%, n = 5). Since human research studies are often limited by sample sizes, we focused on large effect sizes (Cohen’s d > 1.2) [65] and 95% confidence intervals when considering conclusions drawn from statistical tests.cECs constitute 0.0001–0.01% of peripheral blood mononuclear cells [40]; however, flow cytometric detection requires the targeted cell frequency to be above 0.01% [72]. Therefore, we included CD34^+^ immunomagnetic isolation [36] to enrich cPCs from the peripheral blood and increase cEC frequency for the subsequent VEGFR quantification. To exclude dead cells and platelets, we first selected (gated) large cells, using 6 µm phycoerythrin (PE) calibration beads as a size reference (Fig. 1A). Debris and aggregates were excluded to reduce noise from the signal (Fig. 1B). Our results confirmed prior work showing that CD34^+^ cPCs also express CD31 (Fig. 1C) [39, 59, 63, 68]. Finally, we separated cECs from cPCs with an endothelial-specific marker, CD146 [42]. The enhanced cEC frequency was 0.98 ± 0.29% (mean ± SEM, n = 23) in the CD34-enriched flow samples (Fig. 1D).

Subsequent data analysis focused on three goals: (1) using frequency histograms to establish plasma membrane VEGFR concentrations on single cECs and cPCs, (2) using factorial ANOVA and effect size (Cohen’s d) to identify correlations between subject characteristics (sex, age, race, and menopausal status) and the respective median concentrations of VEGFRs on cECs and cPCs, and (3) adapting a mixture modeling approach, established for quantitative flow cytometry [80] to characterize VEGFR heterogeneity in cECs.

### cPCs and cECs Exhibited Heterogeneous Plasma Membrane Expression of VEGFRs

**Fig. 2:**
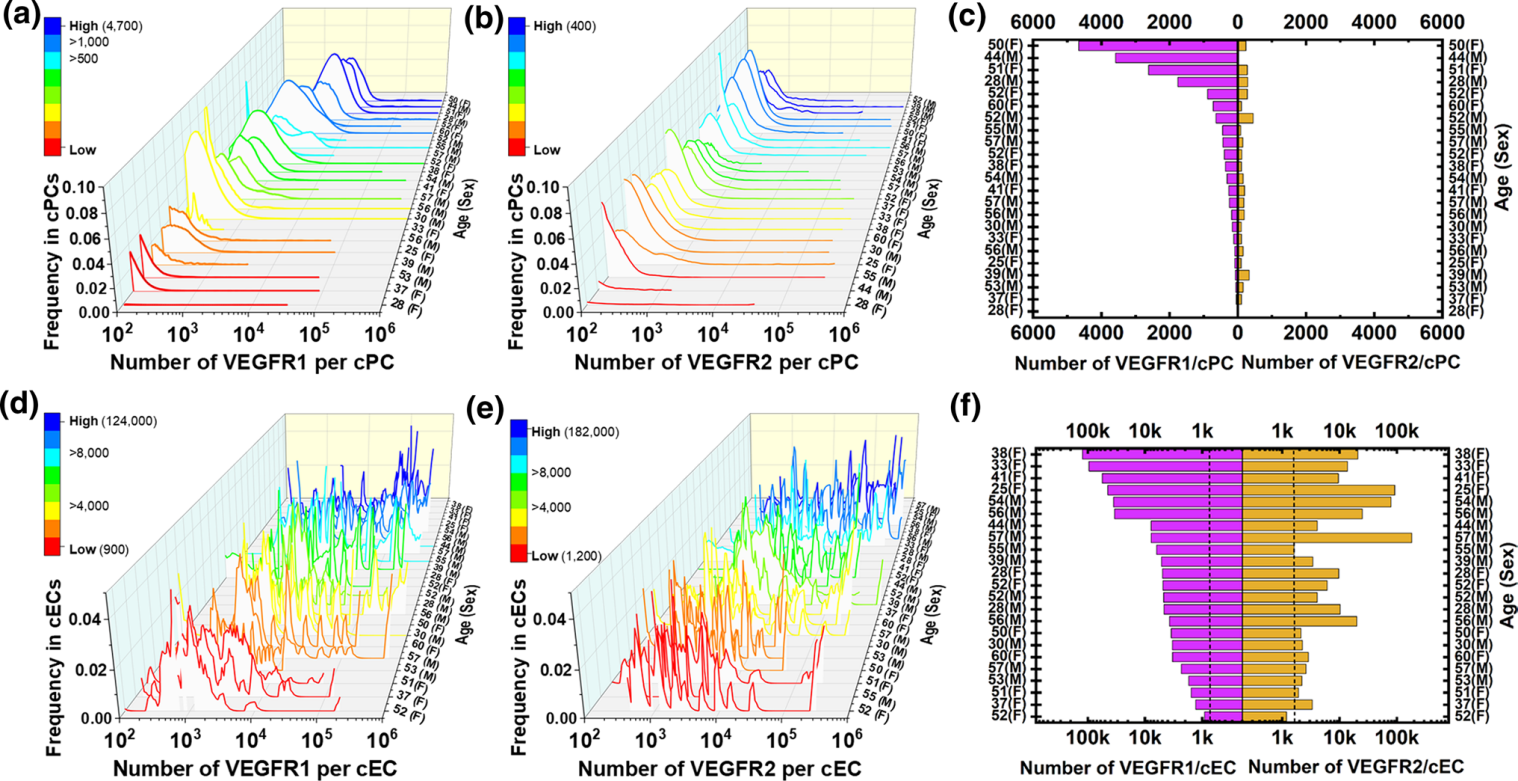
Quantitative cell-by-cell variations of VEGFR expression by CD34 + CD31 + cPCs and CD34 + CD31 + CD146 + cECs in 23 healthy blood samples. The histograms are ranked by median VEGFR concentrations from low to high (color maps). **A** Cell-by-cell distributions of VEGFR1 and **B** VEGFR2 on cPCs in 23 individuals’ blood (age 46 ± 11, 11 females and 12 age-matched males). **C** Median levels of VEGFR1 and VEGFR2 on cPCs were extracted from the frequency histograms. **D** Frequency histograms of VEGFR1 and **E** VEGFR2 on cECs in 23 individuals’ blood. **F** Median levels of VEGFR1 and VEGFR2 on cECs were extracted from the frequency histograms. Dashed lines indicate the previously reported ensemble averages of VEGFR1 and VEGFR2 concentrations of human umbilical vascular endothelial cells, in vitro (800 VEGFR1s and 1800 VEGFR2s per HUVEC). Subject codes are formatted as age (sex)

We quantified the numbers of VEGFR1 and VEGFR2 molecules on the plasma membranes of cECs and cPCs in a cell-by-cell manner and plotted them on log-scale histograms as previously described [16, 80] (Fig. 2A, B, D, E). Median VEGFR concentrations and interquartile ranges (IQRs) of these distributions were calculated and reported, along with subject age, sex, and race, in Suppl. Table 1. It should be noted that the threshold for specific binding of human VEGFR antibodies to the cell membrane is 200–500 VEGFRs/cell on average [18]. Therefore, VEGFR1 plasma membrane expression on cPCs was detected in only the 30% of the subjects who were (post)menopausal females, and VEGFR2 plasma membrane expression on cPCs was not detected. On cECs, VEGFR1 and VEGFR2 plasma membrane expression was detected in all subjects.

The varied plasma membrane VEGFR concentrations across the individuals were quantified and reported as the median of VEGFR distributions: VEGFR concentrations ranged from 10 to 4700 VEGFR1s per cPC (Fig. 2C), 10 to 400 VEGFR2s per cPC (Fig. 2C), 900 to 124,000 VEGFR1s per cEC (Fig. 2F), and 1200 to 182,000 VEGFR2s per cEC (Fig. 2F). Together, these data illustrate the inter-subject variability of VEGFR concentrations on cECs and cPCs across healthy individuals.

### VEGFR Plasma Membrane Localization in Healthy Subjects is Associated with Age-Sex Interaction Effects

Age [44], sex [64], race [55], and menopausal status [49, 58] can alter angiogenic capacity by shifting the balance between anti- and pro-angiogenic factors, influencing individual susceptibility to vascular diseases and cancers. We examined whether these characteristics correlated with intersample VEGFR variations in cPCs and cECs. All the females in this study aged above 50 years old self-reported as (post)menopausal, and those below 50 years old were self-reported as peri/premenopausal; menstrual status was thus considered an age-sex interaction effect. We used the age of 50 years old as a cutoff for the statistical interpretation of the effects of age on median VEGFR concentrations of cPCs and cECs from the healthy females and those from age-matched males.

We found that menstrual status was significantly correlated with cPC and cEC VEGFR concentrations. We detected six statistically significant sex-by-age interactions (p < 0.05), as summarized in Table 1. The median VEGFR1 concentrations of cPCs were higher in menopausal/postmenopausal females (n = 6) than in peri/premenopausal females (n = 5) (p = 0.0014) and age-matched males (p = 0.0054). The median VEGFR1 concentrations of cECs were higher in peri/premenopausal females than in menopausal/postmenopausal females (p = 0.002), age-matched males (28–44 years old, p = 0.008), and older males (52–57 years old, p = 0.0082). Moreover, median VEGFR2 concentrations of cECs were higher in peri/premenopausal females than in menopausal/postmenopausal females (p = 0.0265). None of the three factors (age, sex, and race) were independently associated with VEGFR concentrations. No significant sex-by-race or race-by-age interaction was detected in VEGFR concentrations.

**Table 1:** Significant sex*age group comparisons on VEGFR concentrations of cECs and cPCs based on factorial ANOVA

Sex*age groups	Estimated mean difference (log scale)	95% confidence interval (log scale)	p-value	Effect size (Cohen’s D)
Median VEGFR1 concentration of cPC				
≥ 50 years females *postmenopausal*	2.50	[1.12, 3.88]	0.0014	2.56
< 50 years females *premenopausal*				
≥ 50 years females *postmenopausal*	2.12	[0.72, 3.53]	0.0054	1.97
≥ 50 years males				
Median VEGFR1 concentration of cEC				
≥ 50 years females *postmenopausal*	2.46	[1.04, 3.88]	0.002	2.45
< 50 years females *premenopausal*				
< 50 years females *premenopausal*	2.05	[0.61, 3.5]	0.0082	1.75
≥ 50 years males				
< 50 years females *premenopausal*	2.38	[0.71, 4.04]	0.008	2.17
< 50 years males				
Median VEGFR2 concentration of cEC				
≥ 50 years females *postmenopausal*	1.87	[0.25, 3.49]	0.0265	1.63
< 50 years females *premenopausal*				

Median values of VEGFR distributions were compared across 23 healthy samples. Six significant comparisons were identified as p-value < 0.05 with a 95% confidence interval. A Cohen’s D (a standardized effect size) was estimated from the factorial ANOVA. The present study yielded very large Cohen’s D values, ranging between 1.63 and 2.56, indicating that the two groups’ means differed by around 2 standard deviations

To assess the magnitude of the differences between peri/premenopausal females, menopausal/postmenopausal females, and males, we computed effect sizes (Cohen’s d) independent of the sample size. As a general guide, effect sizes of 0.3, 0.5, 0.8, 1.2, and 2 correspond to mild, moderate, large, very large, and huge effect sizes, respectively [65]. A large effect size is desired in biomarker research because a biomarker is useful when biomarker levels of two study groups are far apart. In the present study, the effect sizes were “very large”, ranging between 1.63 and 2.56, indicating that the two groups’ means differed by 1.63–2.56 standard deviations (Table 1). These large effect sizes indicate that inter-sample variations of VEGFR plasma membrane localization in healthy subjects are significantly associated with age-sex interaction effects, like menstrual status, and are important considerations when studying VEGFR membrane localization and signaling mechanisms.

### VEGFR1 Plasma Membrane Localization in cPCs is Detected Only in Menopausal/Postmenopausal Females

**Fig. 3:**
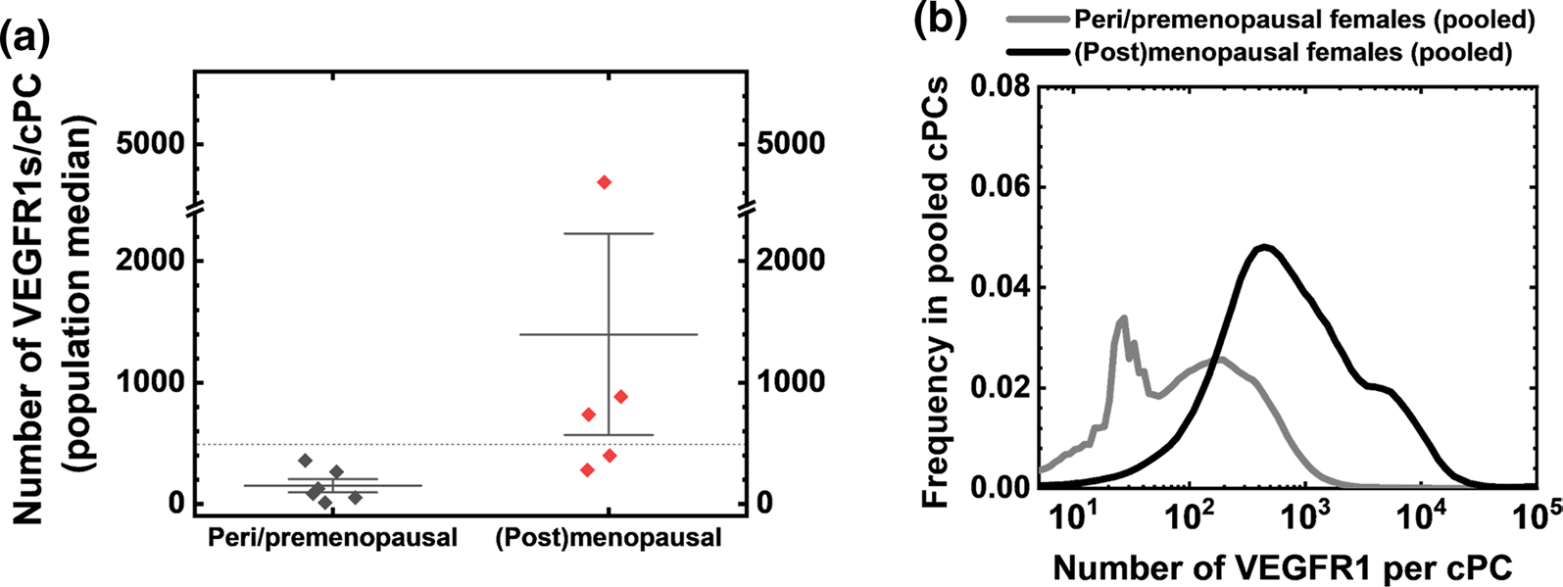
VEGFR1 plasma membrane localization in cPCs is detected in menopausal/postmenopausal females, not in peri/premenopausal females. **A** The numbers of VEGFR1s/cPC of individual female samples are calculated as the population medians of each cell sample (10,000 cPCs per sample). The dashed line indicates the previously established detection threshold (sample median, 500 VEGFRs/cell). The VEGFR1 measurements are under the detection threshold for all the peri/premenopausal females. The error bars represent mean ± SE (p = 0.2). **B** Pooled VEGFR1 measurements represent the difference in the VEGFR1 distribution on cPCs between females over 50 years (n = 5) and females under 50 years (n = 6). The population medians of VEGFR1 concentrations are 88 VEGFR1s/cPC and 650 VEGFR1s/cPC in pooled peri/premenopausal females and menopausal/postmenopausal females, respectively

Increasing evidence has shown that menopause alters angiogenic capacities, as manifested by decreased blood VEGF concentrations, decreased endothelial cell proliferation [58], and increased risks for cardiovascular diseases [44]. Here, our data illustrate that, though the differences in VEGFR2 levels in cPCs were negligible between samples, cPC VEGFR1 distributions were increased (more right-skewed median levels) in menopausal/postmenopausal females compared with those in peri/premenopausal females (Fig. 3A). VEGFR1 plasma membrane localization in cPCs was thus expected to play a more significant role in regulating angiogenesis in menopausal/postmenopausal females than in peri/premenopausal females. Therefore, to isolate the nonpathological variable of menopause, we established separate VEGFR baselines of cPCs for menopausal/postmenopausal females and peri/premenopausal females (Fig. 3B).

We detected a median of 650 VEGFR1s/cPC on the plasma membrane in the pooled menopausal/postmenopausal females’ cPCs, whereas the pooled peri/premenopausal females’ cPCs had a median of only 88 VEGFR1s/cPC (Fig. 3B). We typically describe membranous VEGFR concentrations below 500 VEGFRs/cell as little-to-no, because prior tests of nonspecific antibody binding results in the quantification of between 200 and 500 receptors [18]. Male cPCs also exhibited low-to-no plasma membrane VEGFR1s (Table 2) [21]. Overall, the numbers of VEGFR1 molecules localized to the membrane on cPCs in peri/premenopausal females and males (88–330 VEGFR1s/cPC) were considered negligible. VEGFR2 plasma membrane expression on cPCs was also negligible in all healthy blood samples (median, < 200 VEGFR2s/cPC).

**Table 2:** Descriptive statistics of VEGFR expression baselines in each sex*age group

Subjects	Number of VEGFR1 per cPC		Number of VEGFR1 per cEC		Number of VEGFR2 per cEC	
	Median	IQR	Median	IQR	Median	IQR
Female	240*	610	4300	44000	5100	40000
≥ 50 years, postmenopausal	650	1500	2300	6000	2400	7400
< 50 years, premenopausal	88*	214	25000	122000	18000	91000
Male	265*	580	5100	37000	5400	30000
≥ 50 years	250*	390	4700	54000	5600	68000
< 50 years	330*	2400	5200	13000	4300	9300

Blood samples are pooled based on sex*age interaction categories. Median and IQR values of VEGFR concentrations are extracted from the pooled cell-by-cell VEGFR distributions to represent the healthy levels of VEGFR1 and VEGFR2 on cECs and cPCs in these sex*age groups

*Indicates the values are below the previously established quantifiable threshold (median 500 VEGFRs/cell) (Chen [18], book chapter). Values of VEGFR2 per cPC are not shown because they were all below the 500 VEGFR/cell threshold (Supp. Table 1)

### High-VEGFR cEC Subpopulations are Detected in Peri/Premenopausal Females and Males of All Ages

cECs shed from various tissues exhibit considerable vascular heterogeneity (e.g., different gene expressions, functions, and morphology) [41]. Identifying the normal cEC subpopulations in healthy individuals is crucial to distinguishing abnormal cEC subpopulations shed from diseased vascular beds. To this end, we investigated VEGFR-based cEC subpopulations in healthy individuals, taking into account the significant age–sex interactions (Table 1).

**Fig. 4:**
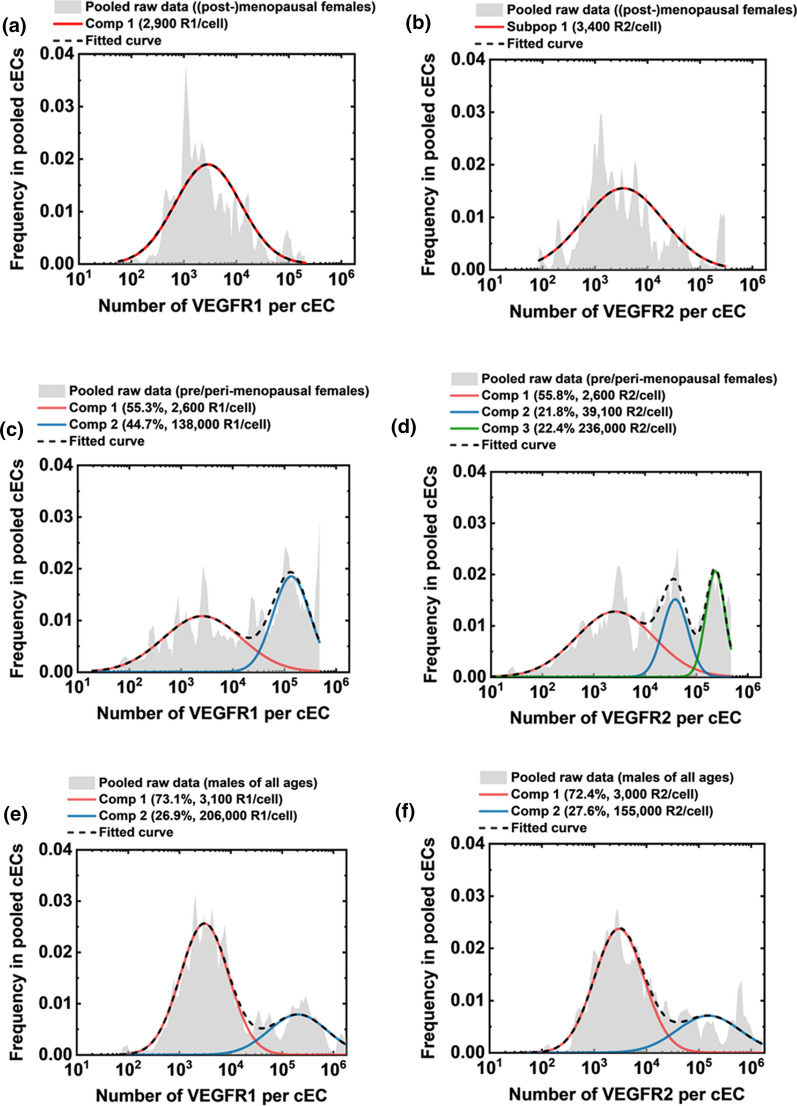
Peri/premenopausal females have prominent VEGFR-intermediate/high cEC subpopulations that menopausal/postmenopausal females (50–60 years) lack. Inspired by the age-specific differences in the median VEGFR concentrations on cECs, we separately pooled the VEGFR measurements from **A**, **B** Menopausal/postmenopausal females (n = 5) and **C**, **D** Peri/premenopausal females (n = 6) to compare the VEGFR distributions between these two groups. Each component (Comp) represents a cEC subpopulation identified via mixture modeling analysis. **A**, **B** In menopausal/postmenopausal females, cECs expressed 2900 VEGFR1 and 3400 VEGFR2 per cell (median of cell populations). **C** In peri/premenopausal females, 44.7% cECs made up VEGFR-intermediate/high subpopulations that expressed 138,000 VEGFR1/cell. **D** The peri/premenopausal females showed three cEC subpopulations based on median VEGFR2 concentrations: 55.8% cECs had 2600 VEGFR1/cell, 21.8% had 39,100 VEGFR2/cell, and 22.4% had 236,000 VEGFR2/cell. **E** Since no age-related difference was statistically identified among males, we pooled cEC measurements from males of all ages (n = 12) and observed two cEC subpopulations based on VEGFR1 densities: 73.1% cECs had 3100 VEGFR1/cell and 26.9% had 206,000 VEGFR1/cell. **F** We observed two cEC subpopulations based on VEGFR2 concentrations: 72.4% cECs had 3000 VEGFR2/cell and 27.6% had 155,000 VEGFR2/cell. These cEC subpopulations in healthy individuals were stratified and quantitatively characterized by their median VEGFR concentrations via adapting an established mixture modeling

We found that cECs in peri/premenopausal females consisted of two VEGFR populations: VEGFR-low and VEGFR-high, while the menopausal/postmenopausal group only possessed the VEGFR-low cEC population (Fig. 4). The VEGFR-low and VEGFR-high cEC subpopulations were identified mathematically via a previously established Gaussian mixture modeling method [17] and quantified by the median VEGFR concentrations: (1) cECs in menopausal/postmenopausal females were generally VEGFR-low, presenting ~ 3000 VEGFR1s and ~ 3000 VEGFR2s per cell (Fig. 4A, B); (2) about half of the cEC population in the peri/premenopausal female group presented one-to-two orders of magnitude more VEGFRs, having 138,000 VEGFR1s/cell and 39,000–236,000 VEGFR2s/cell (Fig. 4C, D); and (3) a quarter of the cEC population in males presented high VEGFR numbers, having 206,000 VEGFR1s/cell and 155,000 VEGFR2s/cell (Fig. 4E, F). Table 2 summarizes the descriptive statistics of VEGFR concentrations in cPCs and cECs, including the medians and IQRs, for females above 50 years old and under 50 years old and for age-matched male groups.

## Discussion

Our approach, which quantifies VEGFR protein expression on circulating angiogenic cells, has the potential to serve as a valuable tool for VEGFR-based diagnosis and prognosis. Changes in VEGFR protein expression are effective indicators in several clinical prognoses. For instance, immunohistochemical VEGFR1 overexpression is predictive of decreased overall survival in colorectal cancer patients treated by the anti-VEGF agent bevacizumab [81], and it can also help identify metastasis in ovarian cancer patients [71]. Similarly, VEGFR2 overexpression has been identified as a biomarker of increased tumor vascular density [47, 70]. By offering this quantitative and non-invasive approach, our work could expedite the translational application of VEGFR biomarkers.

Sex differences in endothelial cells and angiogenic regulators are known to contribute to sex-specific mechanisms in angiogenesis [64]. Our noninvasive, vascular cell-targeted biomarker approach has revealed significant, quantifiable differences in VEGFR1 and VEGFR2 plasma membrane localization in cECs and cPCs between menopausal/postmenopausal females, peri/premenopausal females, and males of all ages. Our findings, for the first time, provide quantitative insights into how sex-age interactions influence VEGFR plasma membrane localization in circulating angiogenic cells. Most importantly, we established separate healthy baselines for VEGFR expression on the plasma membranes of cECs and cPCs for menopausal/postmenopausal females, peri/premenopausal females, and males of all ages. This presents a necessary first step towards precision medicine and supports future investigations into the predictive values of circulating vascular cells in VEGFR-driven prognoses.

**Fig. 5:**
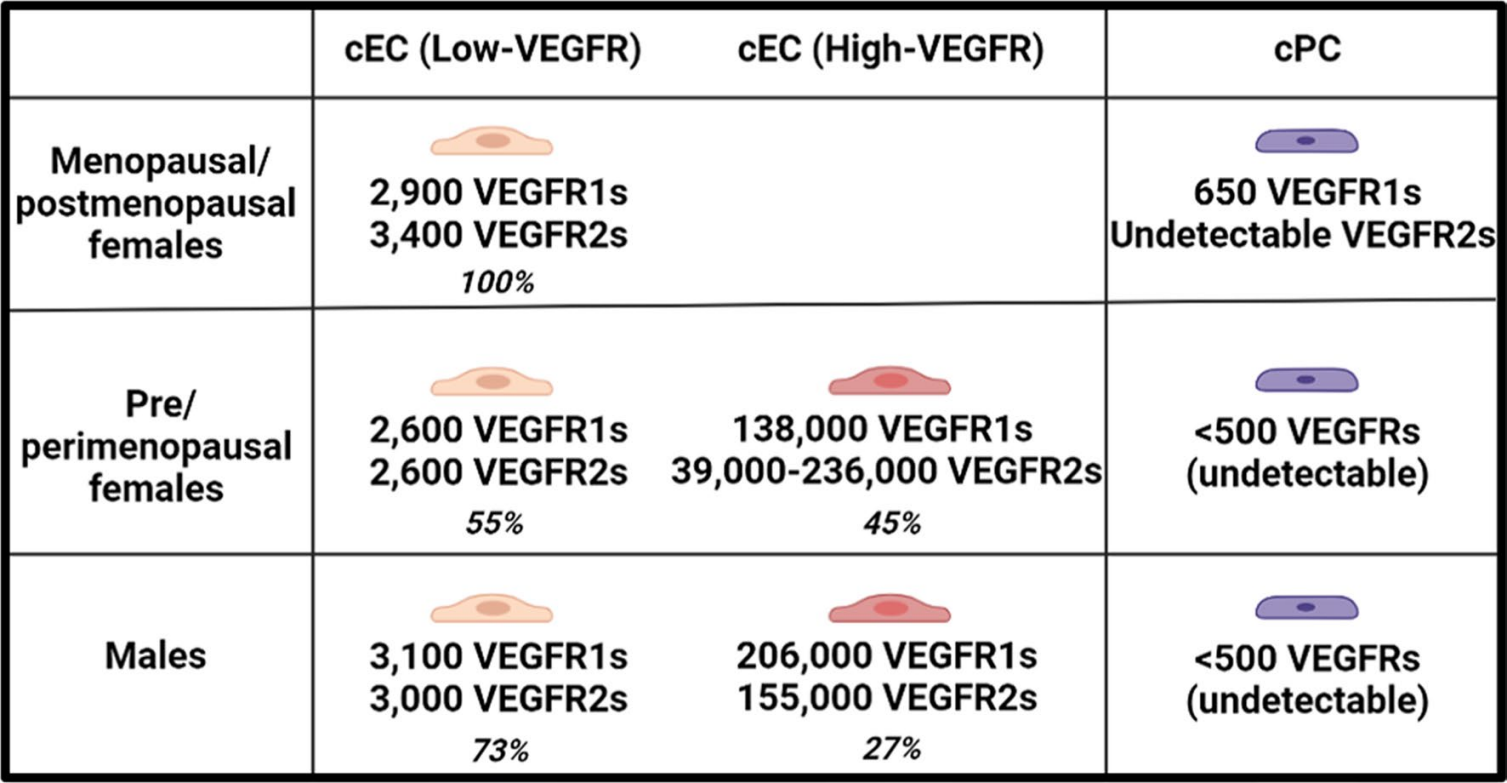
Hypothetical cPC and cEC population compositions in healthy females and males

Different cEC subpopulations may be attributed to aging-dependent sex differences in angiogenesis. Notably, menopausal/postmenopausal females’ cECs homogeneously exhibit low plasma membrane VEGFR levels, whereas peri/premenopausal females’ cECs are more heterogeneous, consisting of ~ 50% VEGFR-low and 50% VEGFR-high cECs [26] (Fig. 4A–D). Males possess a smaller fraction (27%) of VEGFR-high cEC subpopulations, which may originate from male-prone angiogenic processes, such as visceral fat expansion–induced adipose angiogenesis [57]. Together, our data suggest distinguishable membranous VEGFR distribution patterns in cEC subpopulations of different sex-age groups (Fig. 5).

One possible source of the VEGFR-high cEC subpopulations in peri/premenopausal females is the turnover of vascular endothelial cells due to estrogen-enhanced angiogenesis during menstruation [48]. During the menstrual cycle, angiogenesis forms new endometrial endothelium [50, 61]; the high-VEGFR cECs are potential non-invasive proxies for studying endometrial angiogenesis [62]. Our non-invasive method has the potential to detect the upregulation of VEGFR2 in endometriotic vessels [11] by quantifying the VEGFR2 molecules on cECs obtained from women with endometriosis. In addition, compared to soluble biomarkers such as DNA, mRNA, metabolites, and soluble proteins (such as VEGF and soluble VEGFR), our cell-based VEGFR measurements offer a more direct representation of endothelial cell phenotypes in the tissue origin. Thus, measuring the VEGFRs on the membrane is more pertinent to endometriotic angiogenesis than measuring soluble VEGFRs, which did not show a correlation with the occurrence of endometriosis [76].

Future studies using single-cell RNA sequencing can confirm cEC origins: this has been performed successfully on vascular cells of brain [29, 37], lung [29, 37], heart [37], and endometrium [41]. Although the origins of the VEGFR-high cECs in healthy males and peri/premenopausal females need to be further identified, cECs shed from diseased vasculature and cancers are expected to outnumber their normal counterparts (Table 3), allowing for precise identification of diseased cEC subpopulations via mixture modeling.cPCs play an important role in vascular regeneration, and the distinct VEGFR1 membrane localization on menopausal/postmenopausal cPCs suggests a menopause-specific angiogenic mechanism. This is further supported by the fact that VEGFR1 membrane localization is undetectable in peri/premenopausal females and males of all ages. Menopause is a major sex difference that depends on age and correlates with increased risk for vascular diseases and cancers, [27] so the presence of VEGFR1^+^ cPCs in menopausal/postmenopausal females begs the question of its implication on cancer risk. We propose further studies of the menopausal/postmenopausal cPC connection to cancer risk for three reasons: (1) VEGFR1^+^ cPCs are linked to tumor vascularization and metastasis [30], (2) VEGFR1 signaling on cPCs promotes cPC recruitment to activated tumor blood vessels, giving rise to endothelial cells or myeloid cells that produce essential vascular growth factors like VEGF-A [1, 45], and (3) VEGFR1^+^ cPC clusters make up pre-metastatic sites, which permit the attachment of circulating tumor cells and endothelial progenitor cells to form metastases [38]. Further studies are needed to determine whether the increased VEGFR1 plasma membrane localization in cPCs is associated with the higher risk of vascular diseases and cancer seen in menopausal/postmenopausal females [14, 31].

**Table 3:** Reported cEC counts in disease studies of vascular disorders and cancers

Patient type	cEC counts in disease (range)	cEC counts in healthy (range)	cEC markers	References
Vascular disorders				
Acute myocardial infarction	52/ml (28–81.5)	10.5/ml (6–16.5)	CD146^+^, CD31^+^, vWF^+^	Wang et al. [87]
	10.6/ml (5.8–14.5)	2.75/ml (1.6–3.9)	CD146^+^	Lee et al. [88]
	4.9/ml (3.6–8.4)	1.0/ml (0.5–1.7)	CD146^+^	Makin et al. [89]
Pulmonary hypertension	33.1 ± 1.9/ml	3.5 ± 1.3/ml	CD146^+^	Bull et al. [90]
	56.7/ml (26.9–119.8)	3.6/ml (1.3–10.4)	CD146^+^	Smadja [91]
Acute ischemic stroke	15.5/ml (10.8–20.7)	2.7/ml (1.6–3.7)	CD146^+^	Nadar et al. [92]
Diabetic kidney disease	16.7 ml (9.7–28.1)	5.5/ml (4–7.3)	CD45^dim^, CD31^+^, CD146^+^	Farinacci et al. [93]
Deep vein thrombosis	5000/ml (300–45,000)	100/ml (0–300)	CD146^+^, CD31^+^, CD34^+^, CD144^+^	Alessio et al. [94]
COVID-19	46/ml (32–89)	14.5/ml (9–20)	NA	Nizzoli et al. [95]
	ICU: 49/ml (24–103) Non-ICU: 18/ml (6–70)	< 20/ml	CD146^+^	Guervilly et al. [96]
Cancers				
Progressive cancers (mixed types)	438 ± 65/ml	121 ± 16/ml	CD146^+^, CD31^+^	Beerepoot et al. [97]
Glioblastoma	59.3/ml (0–954)	NA	CD146^+^, CD105^+^, CD45^−^	Vaz Salgado et al. [98]
Metastatic breast cancer	4.25/ml (0–192)	NA	CD146^+^, CD105^+^, CD45^−^	Bidard et al. [99]
Metastatic colorectal cancer	5/ml (0–280)	NA	CD146^+^, CD105^+^, CD45^−^	Rahbari et al. [100]

cEC counts are described by either median (range) or mean ± SD, depending on the format in the original reports

## Conclusion

Both cECs and cPCs are accessible proxies that provide quantitative molecular insights into VEGFR plasma membrane localization and signaling in hosts’ blood vessels. This work provides a standardized, non-invasive method and baseline data for future studies to quantitatively profile VEGFR plasma membrane localization in cECs and cPCs from patients with angiogenic disorders (e.g., cancers, obesity, and cardiovascular diseases). Utilizing our findings as a baseline, future studies may show that VEGFRs on cECs and cPCs are predictive biomarkers for stratifying patients with vascular dysfunction for more effective vascular-targeted strategies. Our findings emphasize the importance of incorporating the biology of sex and age differences into our paradigms for angiogenesis research. Our approach enables accessible, quantitative, and standardizable protein biomarker analyses that are much needed to advance predictive vascular biomarker development.

## Methods

### Human Blood Samples

Human peripheral blood samples were collected from 23 healthy participants (45.6 ± 11.1 years old, 11 females and 12 males). A verbal screening was performed to gather information from the individuals. This information included their age, menopausal status, as well as any prior diagnosis of cardiovascular diseases and cancers. All the females over 50 years old were menopausal/postmenopausal and the females under 50 years old were peri/premenopausal in this study. None of the participants had been diagnosed with cardiovascular diseases or cancers. None reported hormone replacement therapy history. The blood samples were processed under institutional review board–approved protocols at the BioreclamationIVT facility (now BIOIVT, Westbury, NY). The blood samples were tested following FDA regulations and found negative for HBsAg, HIV 1/2 Ab, HCV Ab, HIV-1 RNA, HCV RNA, and STS. The blood samples were stored in K_2_EDTA, shipped with ice packs on the day of blood draw, and received and analyzed within 24 h.

### Bulk RBC Lysis

Upon receipt, nine parts of diluted 1× RBC Lysis Buffer (BioLegend, Cat #420301) were added to one part of whole blood (i.e. 20 ml) and incubated for 15 min at room temperature, followed by centrifugation (350×*g*, 4 °C, for 5 min). Lysed whole blood cells were washed twice with 10 ml stain buffer (PBS supplemented with 0.5% bovine serum albumin, 0.09% sodium azide, and 2 mM EDTA), centrifuged (350×*g*, 4 °C, for 5 min), and resuspended in 1 ml stain buffer for the subsequent immunomagnetic enrichment.

### CD34^+^ Cell Enrichment

To every 0.5 ml of lysed RBC cell suspension (approx. 2 × 10^7^ PBMCs from 10 ml whole blood [54]), 25 μl of DSB-CD34 biotinylated antibody was added. The DSB-CD34 biotinylated antibody (0.4 mg/ml) was prepared by conjugating purified human CD34 antibody (clone My10 or 581, BioLegend) to DSB-X biotin (DSB-X Biotin Protein Labeling Kit, Cat No. D-20655) per the manufacturer’s protocol, and according to our prior work [33, 34, 36]. The mixture was gently pipetted to mix and then incubated on ice for 20 min. After a washing step in 2 ml of ice-cold stain buffer, it was centrifuged at 350×*g* at 4 °C for 10 min without braking. The cell pellets were resuspended in 1 ml stain buffer and transferred to a 1.5-ml Eppendorf tube.

Next, 75 µl FlowComp Dynabeads coated with streptavidin (Invitrogen, cat no. 11061D) were vortexed thoroughly (approx. 10 s), washed twice with 1 ml stain buffer, and resuspended in 75 µl of ice-cold stain buffer. Thereafter, 75 µl bead solution was added to the 1 ml DSB-CD34 labeled cell suspension. The bead-cell mixture was incubated on a rotator (approx. 6 rpm) at 4 °C for 20 min. Next, the bead-cell mixture was diluted at a 1:1 ratio with stain buffer and placed in a magnetic separator (DynaMag-5) for 2 min. After removal of the bead-free, unbound CD34-negative cell suspension by careful pipetting, CD34^+^ cells remained on the wall of the tube due to the magnetic field. The tubes were then removed from the magnetic separator so that beads were released from the magnet walls. The bead-cell mixture was collected, resuspended in 1 ml stain buffer, and placed back into the magnetic separator for at least two more washing steps, after which all the beads were combined in a new Eppendorf tube with 1 ml of biotin-rich release buffer (FlowComp) and incubated on the rotator at 4 °C for 10 min. By pipetting the cell suspension 10–15 times, the CD34^+^ cells were detached from the beads by biotin-streptavidin competition. Then the tube was again placed in the magnetic separator. After 2 min, bead-free CD34^+^ cells in the supernatant were carefully collected and transferred to a 5-ml polystyrene FACS tube (BD Biosciences, New Jersey). Cells were concentrated via centrifugation at 400×*g* for 5 min at 4 °C. Cell-free supernatant was decanted. Lastly, the CD34^+^ cells were resuspended in 200–400 µl stain buffer and kept on ice until flow cytometry immunostaining.

### Immunostaining and Flow Cytometric Acquisition of CD34^+^ Cells

Enriched CD34^+^ cells were aliquoted into 20 µl/FACS tubes and stained with APC-conjugated CD146 and APC-Cy7-conjugated CD31 antibodies per the manufacturer’s recommendation (5 µl/test, BioLegend). Either phycoerythrin (PE)-conjugated VEGFR1 (R&D Systems) or PE-conjugated VEGFR2 (BioLegend) was added to each test tube at the saturating concentration (14 µg/ml) [34]. Samples were incubated in the dark for 40 min at 4 °C, washed twice with 2 ml of stain buffer, centrifuged at 400×*g* at 4 °C for 5 min, and resuspended in 100 µl of stain buffer. To assess cell integrity, 2 µl of Sytox Blue was finally added to each tube as a liquid drop-in. Corresponding fluorescence-minus-one (FMO) controls were used for evaluation of the nonspecific binding of monoclonal antibodies to identify the positive/negative boundary for each fluorophore signal [96]. The FMO control contained all the fluorophores in a panel, except for the one being measured.

Flow cytometry was performed on either of two instruments: an LSR Fortessa (BD) or a Beckman Coulter CytoFLEX S Flow Cytometer. Samples were vortexed immediately prior to placement in the flow cytometer. Prior to sample acquisition, PE voltage settings were finalized and Quantibrite PE beads (BD, cat. no. 340495) were collected. Flow cytometric data analysis was performed using FlowJo analytical software or Kaluza analytical software.

The PE fluorophore offers several advantages, such as its high photostability, pH independence, and small size [12, 28]. More importantly, due to its 1:1 protein/fluorophore ratio, the number of PE molecules per cell equals the number of PE-conjugated receptors per cell; applying qFlow with PE-conjugated VEGFR antibodies allows absolute quantification of membrane VEGFR levels. The precision and accuracy of quantitative flow cytometry (qFlow) profiling have been rigorously tested [9, 15, 18, 34, 36, 80, 83].

### Quantitative Flow Cytometric Data Analysis

The levels of VEGFR per cell were acquired by converting the PE fluorescence intensity to the number of PE molecules per cell, using Quantibrite PE beads as previously described [15, 18, 33, 34, 36]. Quantibrite PE beads are polystyrene beads conjugated with different densities of PE molecules: low (474 PE molecules/bead), medium-low (5359 PE molecules/bead), medium-high (23,843 PE molecules/bead), and high (62,336 PE molecules/bead). The geometric mean PE values of the respective bead subsets were exported and used to calculate *m* (slope) and *b* (intercept) in linear regression (Eq. 1). Because PE molecules are conjugated with anti-VEGFRs at a 1:1 ratio, this equation can convert the PE readout of cells directly to the number of PE-receptors/cell. A calibration curve was established to translate the PE fluorescence level to the PE quantity.

1 $$\log_{10} \left( {{{PE_{Geomean} } \mathord{\left/ {\vphantom {{PE_{Geomean} } {bead}}} \right. \kern-0pt} {bead}}} \right) = m \times \log_{10} \left( {{{\# PE} \mathord{\left/ {\vphantom {{\# PE} {bead}}} \right. \kern-0pt} {bead}}} \right) + b$$

PE fluorescence intensities were recorded for individual CD34^+^ cells as geometric mean values, which were exported to Excel files. False-positive PE signals, commonly due to cell auto-fluorescence, were collected from PE-FMO samples and denoted as PE background fluorescence. In order to accurately quantify PE-receptor levels, the PE background fluorescence was subtracted from the PE fluorescence of PE-stained samples, using a weighted integral approach (Eq. 2).

2 $$PE_{{absolute}} = PE_{{stained}} \times \left( {1 - \frac{{{{\left( {\sum {PE_{{background}} } } \right)} \mathord{\left/ {\vphantom {{\left( {\sum {PE_{{background}} } } \right)} {\left( {n_{{PE{\text{-}}FMO}} } \right)}}} \right. \kern-\nulldelimiterspace} {\left( {n_{{PE{\text{-}}FMO}} } \right)}}}}{{{{\left( {\sum {PE_{{stained}} } } \right)} \mathord{\left/ {\vphantom {{\left( {\sum {PE_{{stained}} } } \right)} {\left( {n_{{stained}} } \right)}}} \right. \kern-\nulldelimiterspace} {\left( {n_{{stained}} } \right)}}}}} \right)$$

*PE*_*absolute*_ is the number of receptors per cell obtained after subtracting *PE*_*background*_, which was obtained from PE-FMO samples. *PE*_*stained*_ is the unsubtracted receptor level measured in a PE-stained sample. *n*_*stained*_ and *n*_*PE-FMO*_ are the cell numbers collected from PE-stained and PE-FMO samples, respectively.

### Pooling Samples

The lengths of the datasets were set equal to ensure individual datasets contributed equally to the pool. The length of each cPC dataset was set to 10,000 and the length of each cEC dataset was set to 1000. This was done by using the “datasample” function in Matlab. This function did not change the median or IQR values of the VEGFR distributions of each dataset. The equal-sized datasets of peri/premenopausal females, menopausal/postmenopausal females, males above 50 years old, and males below 50 years old were separately pooled and analyzed using Matlab.

### Mixture Modeling Analysis

A previously established Gaussian mixture modeling [17] was applied to identify log-normal subpopulations within each cEC distribution, described by its median and IQR. A detailed description of heterogeneity quantification via this method was provided by Chen et al. [17] Ideally, the optimal number of cell subpopulations is determined by the Bayesian information criterion (BIC) values, where the lowest value indicates the best fit [17, 77]. However, when the number of data points is low, the conventional BIC standard can result in overfitting. Therefore, in this study, instead of relying on the BIC standard, the mixture modeling algorithm was set to identify three possible subpopulations, which were denoted as low-, intermediate-, and high-VEGFR cEC subpopulations. Although there could be more than three cEC subpopulations, our fitting results provided a realistic estimate of the major cEC subpopulations in healthy blood samples.

### Statistical Analysis

The distributions of VEGFR levels (median and IQR) on CD34^+^CD31^+^ cells and CD34^+^CD31^+^CD146^+^ cells were checked using the Shapiro–Wilk test. For variables that are not normally distributed but are right-skewed with skewness > 1.5, log transformation was applied. We first used one-way ANOVA or the two-sample t-test to assess the relationship between VEGFR levels and the three independent factors (sex, race, and age category). Factorial ANOVA was then performed and included the three independent factors and their possible two-way interactions. The predicted least-squares means of the VEGFR levels were obtained at different levels of a factor that was adjusted for other factors in the model. For significant two-way interactions, the least-squares means of a factor for each level of the other factor were obtained with a 95% confidence interval (CI). We also reported significant least-squares means comparison with 95% CI for age by sex groups. All the statistical tests were two-sided at a significance level of 0.05, and statistical analyses were performed using SAS 9.4 (SAS Inc., Cary, NC). A Cohen’s d (a standardized effect size) was estimated from the factorial ANOVA. As a general guide, Cohen’s d values of 0.3, 0.5, 0.8, 1.2, and 2 correspond to mild, moderate, large, very large, and huge effect sizes, respectively [50]. Effect sizes were used to quantitatively compare the results of studies done in a different setting since Cohen’s d is independent of sample size.

## Supplementary Information

Below is the link to the electronic supplementary material.

Supplementary file1 (PNG 156 kb)—**Supp. Table 1** Healthy patients’ demographic information and descriptive data (median and IQR, %cECs).

## Data Availability

The main data supporting the results of this study are available within the paper and in the supplemental materials. The raw datasets are available for research purposes from the corresponding authors at reasonable request. The mixture modeling code is available upon request.

## Abbreviations

VEGFR: Vascular endothelial growth factor receptor
cEC: Circulating endothelial cell
cPC: Circulating progenitor cell
qFlow: Quantitative flow cytometry
PE: Phycoerythrin
