# Supplementary figures and images for: Toward Blood-Based Precision Medicine: Identifying Age-Sex-Specific Vascular Biomarker Quantities on Circulating Vascular Cells
Source: Cell Mol Bioeng. 2023 Jul 6;16(3):189–204. doi: 10.1007/s12195-023-00771-1 (PMC10338416; doi:10.1007/s12195-023-00771-1)

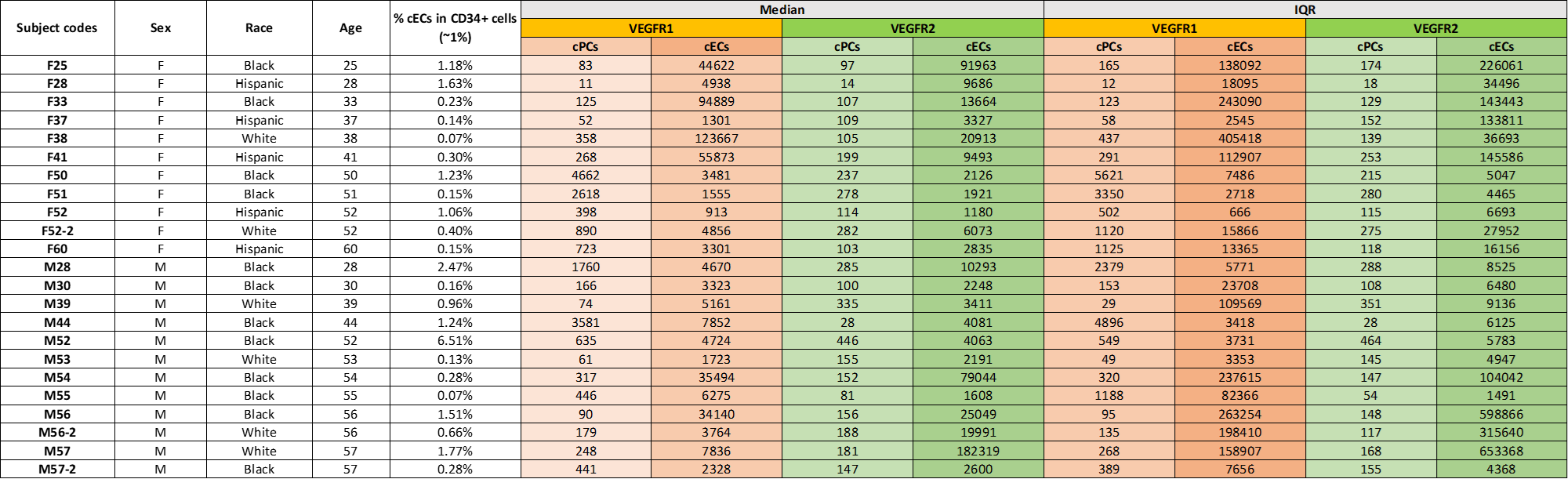

Supplement: Supplementary file 1 — Supplementary file1 (PNG 156 kb)—Supp. Table 1 Healthy patients’ demographic information and descriptive data (median and IQR, %cECs). [file 12195_2023_771_MOESM1_ESM.png]
